# Controlling *Coxiella burnetii* in naturally infected sheep, goats and cows, and public health implications: a scoping review

**DOI:** 10.3389/fvets.2024.1321553

**Published:** 2024-02-15

**Authors:** Raquel Toledo-Perona, Antonio Contreras, Jesús Gomis, Juan José Quereda, Ana García-Galán, Antonio Sánchez, Ángel Gómez-Martín

**Affiliations:** ^1^Microbiological Agents Associated with Animal Reproduction (ProVaginBio), Universidad CEU Cardenal Herrera, Valencia, Spain; ^2^Department of Animal Production and Health, Veterinary Public Health and Food Science and Technology. Universidad Cardenal Herrera-CEU, CEU Universities, Valencia, Spain; ^3^Department of Animal Health, Universidad de Murcia, Murcia, Spain; ^4^Research Group Intracellular Pathogens: Biology and Infection, Universidad CEU Cardenal Herrera, Valencia, Spain

**Keywords:** control measures, Q fever, domestic ruminants, scoping review, one health

## Abstract

Q fever is a worldwide zoonotic disease which domestic ruminants are the main source of infection for humans. This scoping review summarizes the control measures currently available to reduce *Coxiella burnetii* (*Cb*) infection in naturally infected sheep, goat and cattle herds. A total of 28 articles were included in the review. A lack of methodological standardization was noted in the articles analyzed. The results indicated that long-term vaccination in cows reduces bacterial excretion in milk and environmental contamination. In small ruminants, the results of vaccination in terms of efficacy are variable. In goats, there is a reduction in bacterial excretion, unlike in sheep, where a long-term vaccination program is necessary to reduce bacterial excretion. Moreover, the high persistence of viable *Cb* in the environment means that control measures for sheep are needed for several years. The use of antibiotics as a control measure in cows and sheep was not found to reduce excretion. However, the combination of vaccination with antibiotic therapy appears to have positive effects in small ruminants in terms of controlling outbreaks of Q fever. Hygiene and biosecurity measures are the basic means for controlling *Cb* infection on ruminant farms and ensuring public health.

## 1 Introduction

Q fever is a highly contagious zoonotic disease caused by *Coxiella burnetii* (*Cb*). It is endemic globally, except in New Zealand (1), where only one imported human case has been reported (2). Reporting cases of human Q fever is compulsory in 27 European countries and voluntary in France and the UK (3). After a large outbreak in 2007 in the Netherlands (4), the European Food Safety Authority (EFSA) published a scientific opinion on Q fever following a request from the European Commission, which summarized different control measure options in domestic ruminant populations (5). Small ruminants and cattle are recognized as the main sources of human infection (6–8). Under natural conditions, human-to-human transmission is uncommon. As an occupational disease, Q fever mainly affects people in contact with ruminants, such as farmers, slaughterhouse personnel, veterinarians, and veterinary students (9, 10). The main acute symptoms in humans are usually those of a self-limiting, flu-like illness. Clinical complications such as pneumonia, hepatitis, endocarditis, encephalitis, post-Q fever fatigue syndrome, abortions, or premature birth can occur in a chronic Q fever form (5, 11). The disease has a serious impact on ruminant herds because of economic losses experienced due to abortion and loss of milk production (12). In dairy cows, metritis, infertility and mastitis have been described, in contrast to small ruminants, where abortions are the main clinical sign. In general, seroprevalence increases with age, and females with naturally acquired *Cb* infection may show no clinical signs (5). However, goats can remain chronically infected and experience reproductive failure and shed *Cb* in two successive parturitions after a Q fever infection (13).

The implementation of preventive and control measures against *Cb* in ruminant farms is key. There is currently only one inactivated phase I vaccine authorized for small ruminants and cattle. A systematic review and a meta-analysis of Q fever vaccines for small ruminants showed that the vaccine was more effective at preventing the shedding of *Cb* in goats than in sheep (14). Vaccination is used to decrease abortion rates and bacterial dissemination into the environment (9). Antibiotic therapy has been used during the last pregnancy to control clinical outbreaks and to reduce excretion and abortion (15). In addition, biosecurity measures are essential to control Q fever and prevent its dissemination to other herds or humans (5, 16–18). Because of the animal and public health importance of Q fever, our main objective was to evaluate the effectiveness of the current control measures against *Cb* in naturally infected goats, sheep or cattle herds, by means of a scoping review of the scientific literature. Our second objective was to evaluate the type of samples and diagnostic techniques used in naturally infected herds in which control measures against Q fever had been implemented. Thirdly, we aimed to identify information gaps to be filled for future Q fever control and prevention studies in domestic ruminant herds.

## 2 Material and methods

### 2.1 Review protocol, team and expertise

A scoping review protocol was developed a priori by our research team, and pretested before implementation to ensure the reproducibility, transparency and consistency of the articles reviewed. The review team included individuals with multidisciplinary expertise in epidemiology, public health, microbiology, ruminant diseases, animal reproduction, and knowledge synthesis.

### 2.2 Research question

This review was guided by the question, “What Q fever control measures are adopted in naturally infected domestic ruminant herds?” For this study, a scoping review is defined as a type of research synthesis that aims to map the literature on a particular topic or research area. It provides an opportunity to identify key concepts, gaps in the research and types and sources of evidence to inform practice, policymaking and research (19).

### 2.3 Data sources, search strategy, and citation management

The aim of our search strategy was to identify studies published up to 17 November 2021 related to *Cb* infection control measures in naturally infected ruminant herds, following the Preferred Reporting Items for Systematic reviews and Meta-Analyses for Scoping Reviews (PRISMA-ScR) statement (20) and the specific recommendations for its application in veterinary medicine (21).

Our research was carried out in three electronic databases: PubMed^®^, Scopus^®^ and all databases of the Web of Science (Web of Science Core Collection; Current Contents Connect; Derwent Innovations Index; KCI-Korean Journal Database; MEDLINE^®^ Russian Science Citation Index and SciELO Citation Index). The full search strategy was adapted to the syntax of the databases from the following conceptual structure: (“*Coxiella burnetii*” OR “Q fever”) AND (ruminant^*^ OR goat OR caprine OR sheep^*^ OR ovine or cow^*^ OR bovine or cattle) AND (prevent^*^ OR prophyla^*^ OR vaccin^*^ OR shedd^*^ OR control^*^ OR strateg^*^ OR manage^*^ OR outbreak) (Appendix 1). The databases selected had to be comprehensive and cover a broad range of disciplines. No restrictions on the publication date of the studies or literature mapping were applied. This preliminary phase was carried out by three reviewers (RT-P, AG-G, and AC). All records retrieved from the databases were stored using the web-based bibliographic manager EndNote™ (Thomson Reuters, Philadelphia, PA, USA).

### 2.4 Eligibility, inclusion and exclusion criteria

Eligibility criteria defining articles to be included or excluded were developed and applied. Inclusion criteria were applied to publications identified as relevant from RS-1. In our study, control measures were considered as any intervention taken to reduce the impact of coxiellosis on reducing the within-herd and between-herd spread of *Cb* and the risk of transmission from ruminants to humans in naturally *Cb*-infected herds. Any practice aimed at controlling or preventing disease in goat, sheep and cattle was considered, including, but not limited to, vaccinations, antibiotic therapy, biosecurity measures, participation in disease control programs, and their combination. Consequently, only longitudinal observational studies were selected. Included studies had to report the type of samples and diagnostic techniques used. No geographic restrictions were applied. The title and the abstract of the included studies had to be in English, regardless of the language of the rest of the article. The reviews on Q fever in ruminants were considered as an additional source of articles that may not have been detected through the databases used. Gray literature was not processed. Other exclusion criteria considered were articles where the control measure was studied in other animal species different from sheep, goat, and cow or based only on human Q fever cases.

### 2.5 Relevance screening of title, abstract, and data characterization

Citations were analyzed in three stages depending on the title and abstract for subsequent relevance screening and data characterization of full articles. A first screening (RS-1) checked titles and abstracts (when available) to identify the studies that fulfilled the criteria inclusion. Two unblinded reviewers (RT-P and AC) independently checked abstracts (RS-1) and full studies (RS-2). After the RS-1 phase, the complete set of selected studies was fully reviewed (RS-2) for definitive inclusion. The reviewers met throughout the screening process to resolve conflicts and discuss any uncertainties related to study selection (22). When the two reviewers disagreed, a further meeting was held to reach consensus for the definitive inclusion or exclusion of each study.

### 2.6 Data extraction and synthesis

For each eligible article, the data were extracted and summarized in an Excel file. The data extracted and the main conclusions regarding the effectiveness of the control measures applied in each study appears in Appendix 2.

## 3 Results

### 3.1 Search and selection of studies

**Figure 1:**
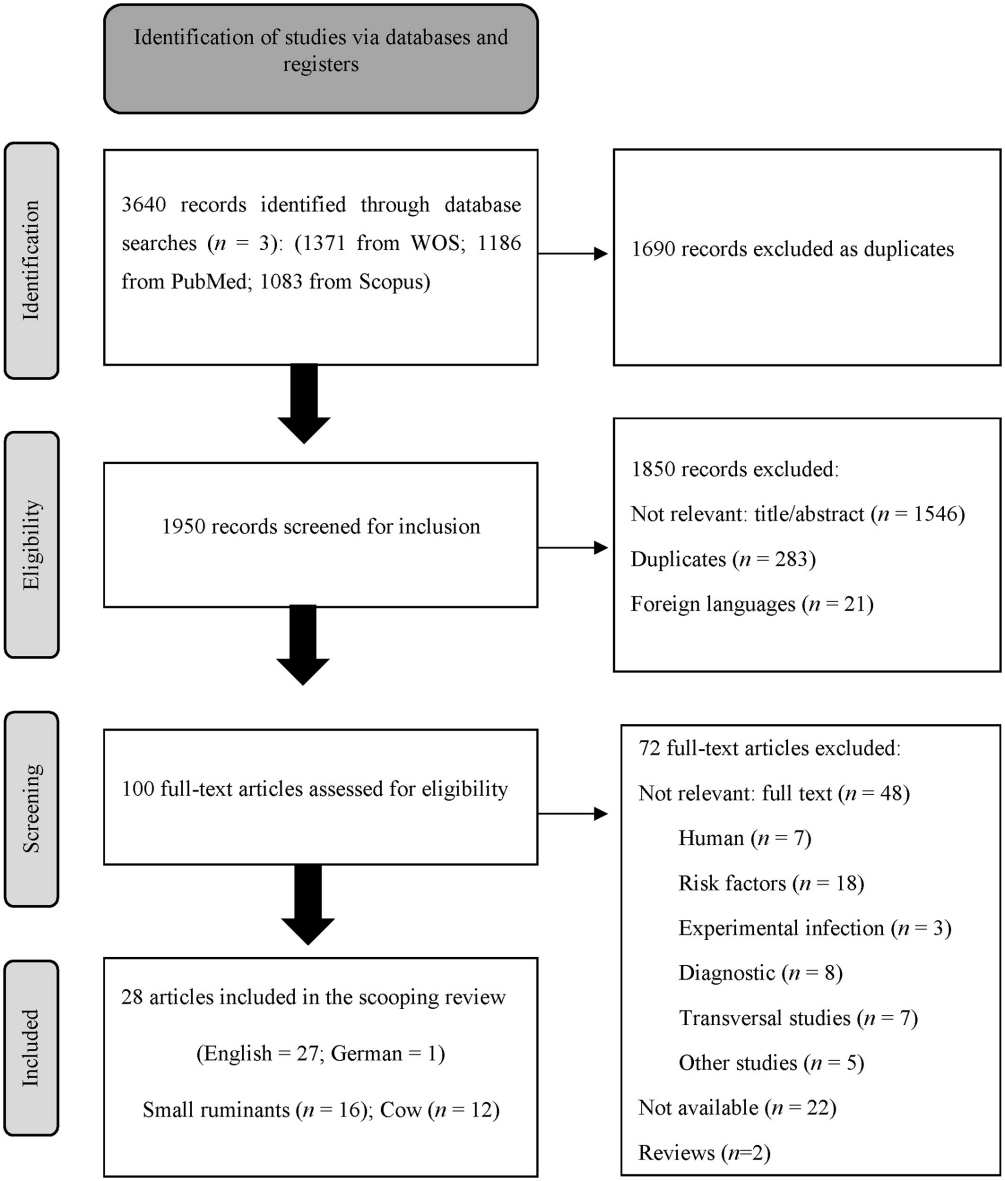
PRISMA chart of the search results of articles by the scoping review process.

A total of 3,640 publications were identified through this exhaustive review of the literature. After removing the duplicates using reference manager software (EndNote™) and manually, a total of 1,950 studies remained in relation to RS-1. Manuscripts in pdf format were obtained through Web of Science (WOS) and Endnote, and unavailable articles were requested by means of the interlibrary loan service of the University of Murcia. A total of 100 studies met our inclusion criteria and were full-text reviewed (RS-2). A total of 28 papers were finally included in our scoping review (Figure 1).

### 3.2 Quantitative description

**Figure 2:**
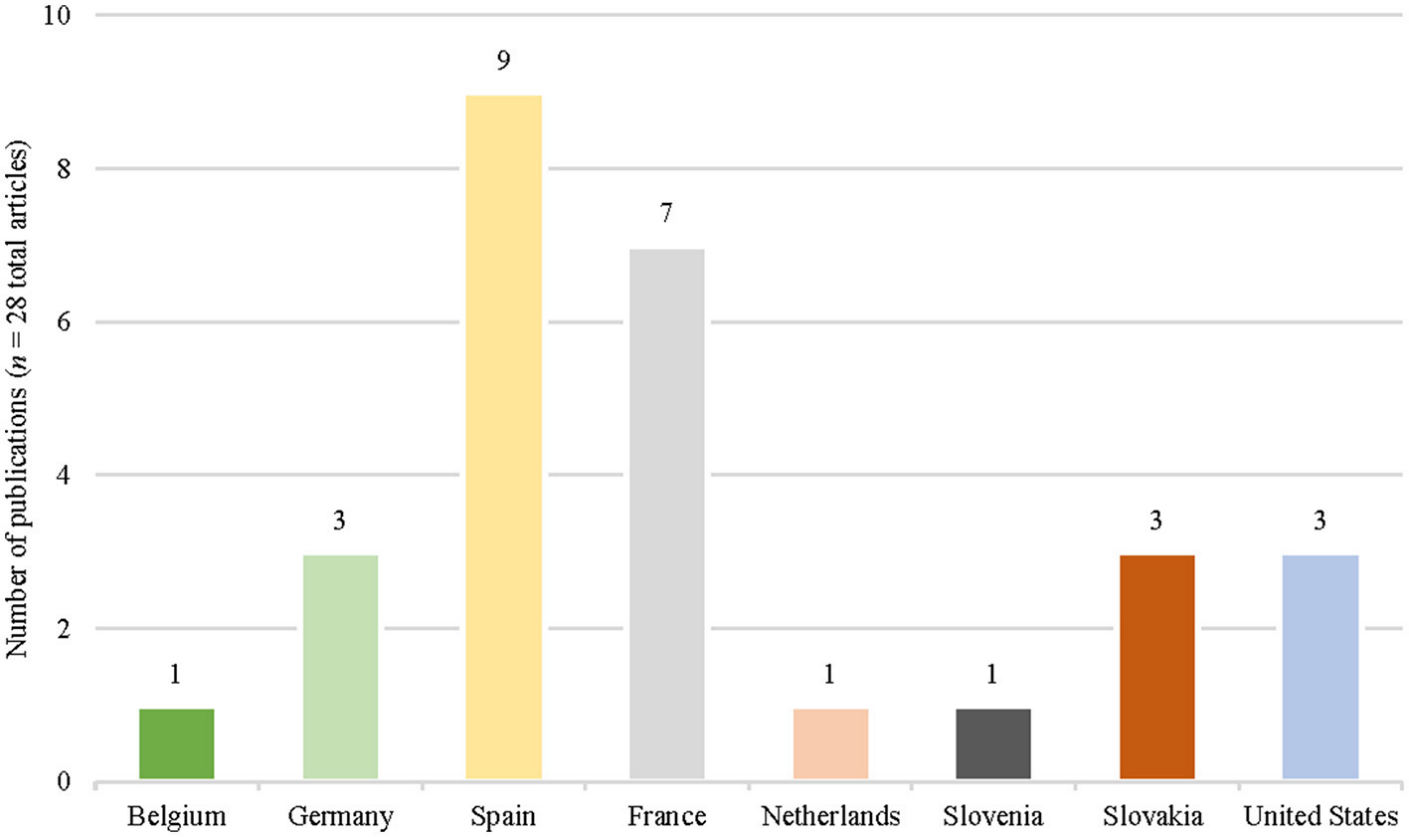
Countries and the number of articles in which the selected studies on controlling Q fever in naturally infected ruminants have been carried out.

**Figure 3:**
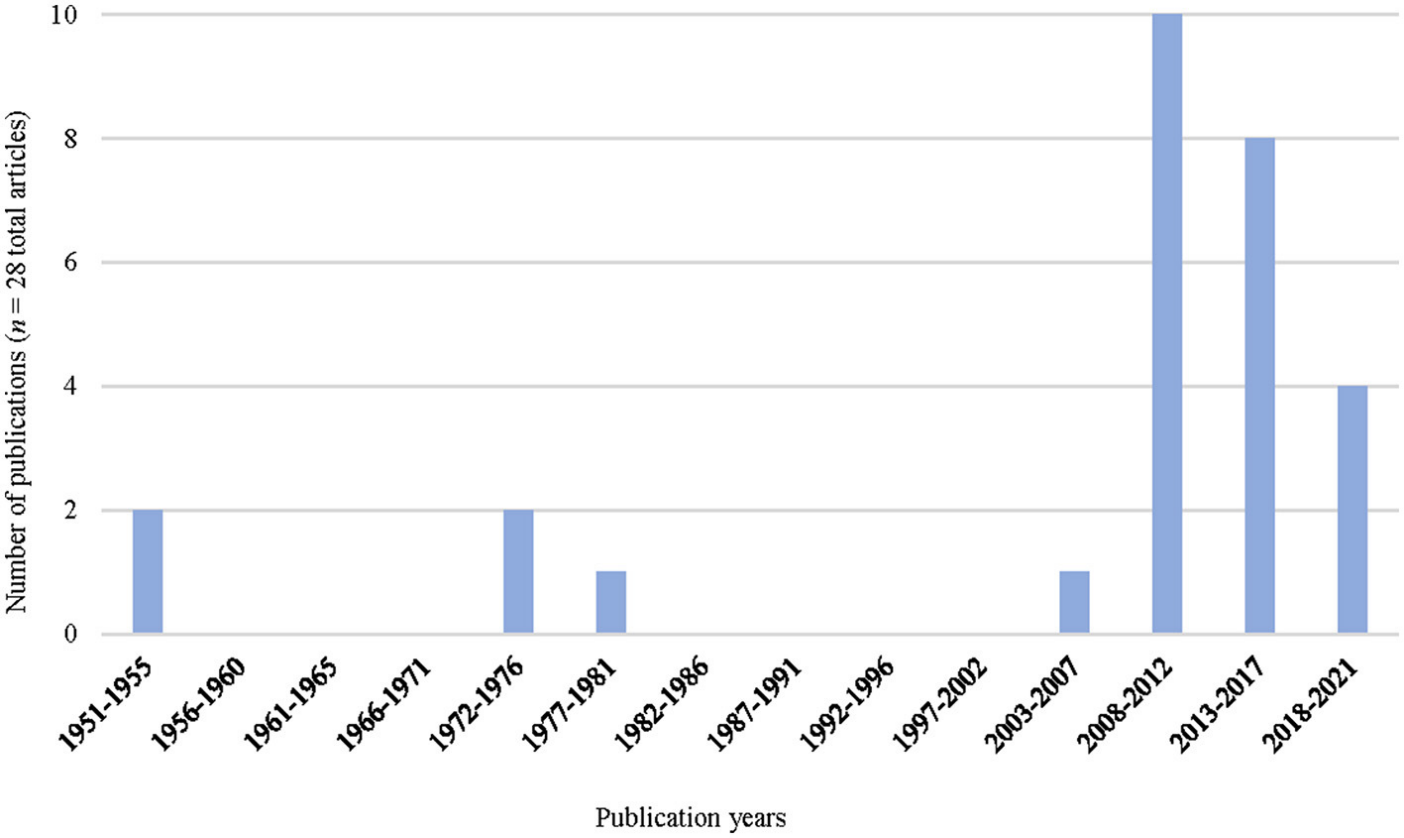
Years of publication of selected studies on the control of Q fever in naturally infected ruminants.

The 28 cited studies concerned studies carried out in 10 different countries (Figure 2): nine were from Spain (32%), and seven from France (25%). Twenty-five of the cited studies (89%) were from Europe. The only country outside Europe with publication studies was the USA, with three studies (11%). The cited studies were published between 1951 and 2021 (Figure 3), and the period between 2009 and 2015 included most (19; 67%) of the studies selected. The years with the most published articles were 2011 and 2014, respectively (8; 29%). All the cited studies were published in English except one, which was published in German.

### 3.3 Description of the studies

Concerning the ruminant species involved, a total of 12 references were on cows (43%), eight on sheep (29%), six on goats (21%), and two were on sheep and goats (7%). Only 18 of the studies (64%) identified the breed of ruminant species. For studies including the breed aptitude of the animals, one study was on a meat goat breed (4%), and 21 were on dairy herds (75%). Only 15 of the studies (54%) described the management system. Of these, all cattle references that described the production system were intensive management, and all small ruminant herds were in a combined indoor/outdoor system (Table 1).

**Table 1:** Description of the study population of included studies.

**Species**	**Breed**	**Aptitude**	**Production system**	**Percentage^a^**	**References**
Sheep	IB, JB	ND	IO	12.50% (1/8)	(23)
	Latxa	Dairy	IO	50% (4/8)	(24–27)
	Préalpes du Sud	ND	IO	12.50% (1/8)	(28)
	ML	ND	ND	12.50% (1/8)	(29)
	ND	Dairy	IO	12.50% (1/8)	(30)
Goat	Alpine	Dairy	IO	16.67% (1/6)	(31)
	Boer	Meat	ND	16.67% (1/6)	(32)
	Saanen (mainly)	Dairy	ND	16.67% (1/6)	(33)
	ND	Dairy	ND	33.33% (2/6)	(34, 35)
	ND	ND	ND	16.67% (1/6)	(36)
Sheep and goat	ML, Texel, Suffolk	ND	ND	50% (1/2)	(37)
	ND	Dairy	ND	50% (1/2)	(38)
Cattle	Fleckvieh, BS, YS, HF	ND	ND	8.33% (1/12)	(39)
	HF	Dairy	Intensive	25% (3/12)	(40–42)
	HF, NM	Dairy	ND	8.33% (1/12)	(43)
	Simenthal	Dairy	ND	16.67% (2/12)	(44, 45)
	ND	Dairy	Intensive	33.33% (4/12)	(46–49)
	ND	Dairy	ND	8.33% (1/12)	(8)

^a^Percentage of articles. BS, Brown Swiss; HF, Holstein-Friesian; IB, Istriana breed; IO, kept indoors in winter conditions and grazing outdoors the rest of the year; JB, Jezersko-Solčava breed; ML, Merino landrace; ND, Not defined; NM, Normande; YS, Yellow Swiss.

Concerning the types of samples used for different studies, up to 16 different sample types were processed. Only three studies (11%) used one type of sample (blood or bulk-tank milk samples). Blood samples were used the most (20; 71%), followed by milk and vaginal swabs. The most frequent combinations were blood, milk and vaginal swab samples (11; 39%) or blood and vaginal swabs (10; 36%). One study processed a combination of eight different sample types (Table 2). According to the diagnostic method used for monitoring *Cb* infection, nine different methods were used. The most common tests used were ELISA and PCR, which were used in 23 studies (82%), a combination of ELISA and PCR used in 14 articles (50%). In six studies (21%), only one diagnostic technique was used (Table 2). All bovine publications (*n* = 12) reported chronic infections. In small ruminants, seven and nine papers focused on an outbreak and chronic situations respectively.

**Table 2:** Type of sample and diagnostic technique used in the cited studies.

**Diagnostic method**	**Type of samples**	**Studies on small ruminants (*n* = 16)**	**Studies on cows (*n* = 12)**
PCR	Vaginal swab	14	4
	Milk	9	4
	Dust	3	1
	Faeces	7	3
	Bulk-tank milk	4	2
	Aerosol	4	1
	Soil	2	0
	Placental	1	2
	Fetuses	1	0
	Blood	1	0
	Manure	2	1
	Bedding	1	0
	Rectal swab	1	0
	Wool	1	0
	Uterine fluid	1	1
	Colostrum	0	1
	Genotyping	Vaginal swab	1	1
Dust		1	1
Bulk-tank milk		1	0
Milk		0	1
Aerosol		0	1
Manure		0	1
ELISA	Blood	8	5
	Milk	3	1
	Bulk-tank milk	1	1
Bacterial isolation	Dust	1	0
	Bulk-tank milk	1	0
Complement-fixation test	Blood	1	4
	Milk	0	1
Guinea-pig test	Milk	0	2
	Placental	0	1
Microagglutination reaction test	Blood	0	2
	Milk	0	1
Mouse inoculation test	Dust	2	0
Vero cell culture	Dust	1	0

### 3.4 Vaccination

In relation to the type of control measure, vaccination, and antibiotic therapy were the most common control measures studied and were used in 24 and 8 studies, respectively. Most of the studies only focused on the effect of vaccination (16; 57%), followed by a combination of vaccination and antibiotic therapy (5; 18%) or only antibiotic therapy (2; 7%). Table 3 describes, for each species, the relation between the control measures implemented and their obtained effect depending on the type of infection.

**Table 3:** Control measures implemented in the articles studied.

**Specie**	**Type of infection**	**Control measure**	**Control excretion^a^**	**Control symptoms^b^**	**No effects reported^c^**
Sheep	Chronic	BM^f^	(27)	-	-
	Chronic	AT	-	-	(29, 48)
	Chronic	VC	(30)	-	(30)
	Chronic	BM^g^ + VC	(23)	-	-
	Acute	AT + VC	(29)	(28)	(46)^d^
Goat	Acute	AT + BM^h^ + VC	(31)	-	-
		BM^i^	(32)	-	-
		BM^j^ + VC	(36)	-	-
		VC	(3, 34)	(38)	-
	Chronic	VC	(33)	-	-
Sheep and goats	Chronic	VC	(38)	-	-
	Acute	VC	-	-	(24)
Cow	Chronic	VC	(23–26, 28)^e^	(8, 42)	(39, 47)
		AT	-	-	(47)
		AT + VC	(48)	-	(49)

^a^Positive bacterial excretion control was reported after implementation of the control measures. ^b^Positive clinic/reproduction effects were reported after implementation of the control measures. ^c^No effects were reported after the implementation of control measures. ^d^The effect of vaccination as a Q fever control measure was not studied. ^e^Only for non-pregnant cows. ^f^Hygiene measures, placenta management. ^g^Hygiene measures. ^h^Control visitor access, herd isolation, hygiene measures, manure management, milk pasteurization, placenta management. ^i^Burial, culling, hygiene measures. ^j^Herd isolation; hygiene measures. AT, antibiotic therapy; BM, biosecurity measures; VC, vaccination.

A total of 24 cited publications used the vaccine as a control measure against *Cb* (13 in small ruminants and 11 in cows). From them, 19 publications (12 on small ruminants and 7 on cows) used an inactivated phase I vaccine (Coxevac; CEVA Santé Animal, France). A total of five articles did not specify the vaccine used, of which four were on cows and one was on small ruminants (Appendix 2). Five of the eight ovine studies did not specify the volume inoculated, whereas three indicated that the dose administered was 1 mL, whereas 2 mL is recommended by the manufacturer.

Previous studies on the dynamics of *Cb* excretion in milk have described the positive effects of vaccination in chronically infected cattle herds during the first year (40, 44) as well as in the reduction of *Cb* elimination through pathways such as colostrum or placenta (45). In a 2-year study, progressive reduction in *Cb* excretion through milk and vaginal fluids was shown in cows after vaccination. Although, manure samples remained positive for at least 18 months (46). Notwithstanding these results, indicated that vaccination in naturally infected lactating cows did not prevent *Cb* excretion in milk or placenta 50 days after vaccination (39).

The effect of vaccination in naturally infected cows was investigated in three of the studies included. A five times lower probability of becoming a shedder in vaccinated non-pregnant cows compared to the placebo group was observed, however pregnant animals presented a similar probability of becoming excretory animals in comparation to those receiving the placebo (43). First studies regarding bacterial shedding in cows described a failure in the bacterial excretion reduction in vaccinated advanced pregnant cows during the first vaccination year. Instead, the authors showed a positive effect with a minor decline in antibodies 3 months postpartum and better transferred immunity from the colostrum to calve (41). During 2 years, reproduction failures were studied in vaccinated pregnant cows with more than three artificial inseminations within the first 150 days of milk production (subfertility) (42). A reduction in the number of cows with reproductive failures after vaccination was observed. In addition, early fetal loss also decreased, however the conception rate at first artificial insemination did not improve. Other authors suggested that alternative techniques, such as skin methods, could be used as a vaccination strategy (50). This method aims to avoid unnecessary revaccinations by evaluating the cellular immune response through intradermal inoculation of the diluted vaccine. One year after vaccination, 80% of the cows were found to have a significant immunity level and the authors concluded that an annual booster was not justified for all animals.

The vaccine effect to control bacteria shedding was investigated in seven small ruminant studies. Sheep studies highlighted the limitations of vaccination to control a *Cb* outbreak in four flocks (37). They described the failure to prevent vaginal shedding during the following lambing season and stressed the importance of monitoring the persistence of antibodies. 2011, during the first vaccination year, there was no significant reduction in the number of vaginal shedders (24). Similarly, the effects of a 4-year vaccination program conducted in heavily infected sheep herds were described (30). Reduced bacterial shedding was observed after the 2^nd^ year in vaginal and milk samples. As found in cattle, environmental samples such as aerosols, remained positive throughout the 4 years of the study. In Belgium, mandatory vaccination on goat farms was used as a Q fever control measure in 124 herds over more than 4 years. Vaccination contributed to the reduction in *Cb* shedding in bulk-tank milk samples. However, this effect did not last more than 2 years after the vaccination (33).

In goats, the number of births and vaccine efficacy was studied showing that young goats shed higher levels of the bacteria through the vaginal route. However, primiparous females had a better immune response to the vaccine than multiparous (34). The authors concluded that, in terms of the goat's age, vaccination should be carried out in yearling goats because of the reduction in *Cb* vaginal shedding after vaccination was higher. In addition, although the vaccine did not prevent infection under high infection pressure, kids or primiparous goats can be protected from clinical symptoms through vaccination (35). Finally, in the Netherlands, the effect of vaccination on bacterial excretion was studied in 13 sheep and goat herds and reported a reduction in the bacterial load in uterine fluid, vaginal mucus and milk, which was most pronounced in primiparous goats (38).

### 3.5 Vaccination and antibiotic therapy

Therapies reported more than 70 years ago described the use of udder infusion and intravenous injection of aureomycin as a potential treatment in naturally infected cows, however no success was found for reducing shedding in milk (47). As in cattle studies, only one study in a naturally *Cb*-infected sheep flock used antibiotics as a single control measure. The results showed that oxytetracycline treatment failed to reduce the probability or duration of bacterial shedding via milk, vaginal or faecal routes (25).

Three studies of sheep and two of cattle investigated the effects of a combination of antibiotics and vaccination in *Cb* excretion reduction. The first studied 22 naturally infected dairy cattle herds which were treated with tetracyclines and vaccinated. They showed a greater reduction in vaginal shedding compared with the effects of both measures separately (48). Two years later, the same authors concluded that vaccination of around 80% of the herd significantly reduced *Cb* milk shedding, while antibiotic therapy was ineffective at reducing bacterial excretion (49). A 3-year study after an outbreak of Q fever in a dairy sheep flock used a combination of antibiotics followed by vaccination as control measures. The use of antibiotics to control symptoms early showed no significant effect. Instead, the number of shedders and the vaginal, faeces, and milk bacterial excretion load decreased during lambing seasons after vaccination. No significant differences in the reduction of *Cb* shedding between the vaccinated and control group were shown. However, the percentage of shedders decreased to minimal levels after two more years of repeated vaccination (26).

A combination of antibiotics and vaccines in sheep reported a reduction in abortions and *Cb* shedding, specifically by the vaginal route (29). On the other hand, authors as Berri (28) found no short-term effect of that combination. Abortions during the next lambing season and an immediate reduction in bacterial shedding were not observed (28). One study of goats described a Q fever outbreak controlled by vaccination and antibiotic therapy with the implementation of biosafety and management measures. These consisted of placenta and manure management, hygiene measures, milk pasteurization, control of visitors' access and herd isolation. A lack of viable *Cb* was achieved 2 months after the last parturition and no new human cases were detected (31).

### 3.6 Biosecurity and hygiene

In 2020, a study evaluated the progression of infection during four lambing seasons in four sheep herds with chronic infection. A reduction in the number of animal shedders in vaginal, faecal, and milk was observed. The control measures implemented consisted of biosecurity and hygienic measures between flocks such as the frequency of manure removal, management of placentas, use of sanitized protective clothes by farm workers, and restriction of visitor access. The authors showed the importance of restricting animal movement and destroying placenta. Despite these results, the persistence of *Cb* in dust at the end of the study evidenced the risk of infection for long periods (27).

Other biosecurity control measures were studied in 17 goat herds (32). Authors described lower environmental contamination with the protective effect of carcass burial combined with temporary hold or quarantine and the restriction of animal movements, and specifically of pregnant females (32). One study described a combination of vaccination and hygiene measures, such as cleaning and disinfection, in a chronically infected flock of sheep with positive results in <2 years, highlighting the value of these biosecurity measures. The positive results highlighted the implementation as a short-term control measure. The authors stressed the importance of manure as a source of environmental contamination with an infection persistence of 3 years (23). Finally, a positive effects of a 2-year control program combining goat vaccination with biosecurity and hygiene measures (no visitors, disinfection and safe disposal of placentas and manure, and keeping females in their last pregnancy stage indoors) was described. Vaginal shedding reduced significantly and reproduction rates quickly improved (36).

## 4 Discussion

### 4.1 Control measures against *Coxiella burnetii* in cattle

To the best of our knowledge, no systematic or scoping review has studied the effects of vaccination on cattle herds naturally infected by *Cb*. A total of nine articles studied the *Cb* vaccine in naturally infected cattle herds as the only control measure, five of which demonstrated a progressive reduction in *Cb* shedding was observed after vaccination in different routes as milk (40, 44) colostrum or placenta (45), and vaginal fluids in long-term vaccinated cows (46). One article described a failure to reduce *Cb* shedding in milk. Unfortunately, there was no control group to compare the level of excretion in vaccinated vs. non-vaccinated cows (39).

The main objectives of Q fever vaccines should be to generate optimal protective immunity to prevent reinfections and shedding of *Cb*. Only one study in 2009 described the optimization of the Q fever vaccine in cows. It was suggested that the immunity level should be assessed before revaccination (50). Three studies showed the importance of the vaccination in cattle's reproductive cycle and showed that the pregnancy state may impact on the vaccine efficacy. This effect has been studied more in cows than in small ruminants, and more in sheep than in goats. The vaccination of pregnant cows showed a positive reproduction impact, as the parameters of subfertility and early fetal loss decreased (42). Not being pregnant or the stage of pregnancy have been described as a factor in the vaccine efficacy. In 2008, an investigation demonstrated that the use of vaccines in non-pregnant cows led to a lower probability of becoming shedders (43), and another study found no reduction in shedding in vaccinated advanced pregnant cows (41).

Firsts results on the use of antibiotics to control milk *Cb* shedding showed no control effects (47). Antibiotic therapy and vaccine are the main control measures used in domestic ruminant-infected herds. This combination showed better results at controlling *Cb* excretion in naturally infected dairy cattle herds (48). The necessary percentage of vaccinated animals in the herds to control *Cb* was described by the same last authors. If the percentage of vaccinated cows in the herd was low or only antibiotic therapy implemented, there was no reduction in *Cb* excretion. These results highlighted the importance of total herd vaccination and the need to reduce the use of antibiotics (49).

### 4.2 Control measures against *Coxiella burnetii* in small ruminants

In relation to small ruminants, a systematic review and a meta-analysis on Q fever-inactivated vaccines analyzed seven different studies published between 1937 and February 2012 (14). Four of these were also included in our scoping review. Although the authors highlighted the limited data available, they showed that the vaccine significantly reduced the risk of excretion through the milk and uterine routes in previously infected goats, which was also found in milk, faeces, and vaginal and placenta secretions from yearling goats. However, no effect of vaccination was found on bacteria excretion in sheep. The number of publications selected in Spain is noteworthy (Figure 2), despite not having experienced significant health problems related to Q fever, as occurs, for example, in the Netherlands. This effect may be more related to the activity of a group of researchers interested in Q fever, than to the census of small ruminants or the appearance of outbreaks. The 28 selected studies included in their introduction, discussion, or both, the public health importance of Q fever (43, 44). Others (31, 36) describe confirmed Q fever infections in humans or suggest (27) using their results to prevent zoonotic risk. Finally, six studies indicate that disease research in animals is motivated by outbreaks in humans (23, 29, 32, 33, 38, 48). Six studies of sheep and goats used vaccination as a single control measure for Q fever. Of these, four studies described the failure to control *Cb* excretion in sheep as a short-term vaccination control measure (30, 33) and no positive control effects on *Cb* excretion (26, 28). Authors pointed out the importance of long-term vaccination and the lack of positive effects of using antibiotics to control clinical signs in animals. However, other studies have indicated a positive effect of controlling *Cb* with a combination of vaccination and antibiotic therapy (29). The use of antibiotics as the only control measure failed to reduce *Cb* excretion (25). Today, it is essential to reduce the use of antibiotics due to the emerging situation of antibiotic resistance, and many alternative antimicrobial strategies have been suggested.

In small ruminant herds, the importance of long-term vaccination is more evident than in cattle. A long-term vaccination program in sheep herds resulted in a bacterial shedding reduction. This study emphasized the persistence of *Cb* in environmental samples, similar to cattle studies (30, 46). The authors suggested the need for long-term vaccination programs, and proposed vaccination as a preventive measure in uninfected flocks or recently infected flocks. Similarly, vaccination did not last more than 2 years in reducing *Cb* excretion from milk in goats (33). The importance of long-term vaccination programs agrees with the findings of Astobiza et al. in sheep herds (30). Regarding the age and number of parities in goats, results have shown the importance of the first vaccination of yearlings (35). Primiparous goats are also essential targets because they shed the most (47), and have a better immune response to vaccination (24). These results are in agreement with the findings by O'Neill et al. regarding the effectiveness of the vaccine depending on age in goats (14). For that reason, vaccination manufacturer's recommendation of females with booster shots is recommended in 280 days in cows and 1 year in goats, and lower than 4 months in the case of sheep after completing the primary vaccination.

Another important factor in Q fever control is the implementation of biosecurity and management measures. Only small ruminant studies have focused on the biosecurity and management measures to control Q fever infection. Two small ruminant studies investigated the combination of vaccination with biosecurity and hygiene control measures. They concluded that this combination could be a short-term control option in sheep flocks in terms of environmental contamination control (23). The combination of vaccination, antibiotics, biosecurity and hygiene measures on a goat farm was effective in controlling a Q fever goat outbreak (31). Similar results were obtained in acute infection in goats using the vaccine and biosecurity measures. The results showed that after a 2-year control program, the infection was controlled (36). Four sheep flocks with chronic infection were studied over four lambing seasons. Despite the positive effects of controlling bacteria shedding, the persistence of *Cb* in dust evidenced the risk of infection over 5 years (27). For that reason, periodical reinfections should thus be considered (31). By controlling environmental contamination through biosafety measures, the authors described success in reducing *Cb* in goat herds where female culling and burial and hygiene measures were implemented. However, they did not recommend the culling of goats as the only control strategy to achieve infection control due to the severity of environmental *Cb* contamination (32), in contrast to the positive effects of culling dairy cows highlighted (46).

### 4.3 Knowledge gaps

Some information gaps were identified in this scoping review that reveal important shortcomings, which in our view need fixing in order to promote standardized Q fever studies. There is a lack of knowledge about the Q fever situation in countries outside Europe and standardized study designs would benefit these regions. Correctly standardized studies are needed regarding the length of the study and the population involved. A lack of consensus was detected to describe the knowledge about the number of vaccinations required for the herd's protection, or even the methods available for assuring a good vaccine response. Due to the intermittent excretion of the bacterium, long-term studies provide a better picture of the dynamics of infection (5).

We also revealed a lack of detailed descriptions both about the populations studied and the animal production systems used. Dairy ruminants are the main Q fever reservoir for humans, maybe because of the intensive systems which involve a higher risk of transmission (51, 52). Despite this, only one article studied infections among a meat goat breed, highlighting the lack of information on controlling Q fever in these breeds. In 25% of the cited studies there was no control group. An included study pointed out that under natural infection conditions, it would be irresponsible to maintain unprotected *Cb*-infected animals due to zoonotic risks (37). The included studies are focused on ruminants naturally infected, not on human cases. Despite not studying specifically human hosts, the zoonotic risk is of primary concern in all the selected articles. Furthermore, a correct diagnosis is best obtained by combining different methods. In four cattle studies, however, the diagnosis was only based on serologic methods. This could have led to the underestimation of the shedding of *Cb*, whereas the PCR assay is a more sensitive indicator of infection and transmission risk (32). In addition, studies based only on PCR could have a lower specificity, since after vaccination, goats may shed DNA but not viable *Cb* in their milk (53). In spite of the above limitations, the observational studies that we analyzed offer useful information on *Cb* naturally-infected domestic ruminant flocks. Such knowledge is critical mainly because of the economic and technical limitations of experimental *Cb* infections, the highly contagious disease, and the risk for public health.

## 5 Conclusion

This scoping review highlights the information available on the control of Q fever in domestic ruminant herds. Vaccination, antibiotic therapy and the implementation of hygienic-sanitary measures on farms were the most common control measures studied. There were few studies reporting a combination of all three control measures, and, in general, all the possible biosecurity measures available to the herds were not implemented. The studies included in our review demonstrated the importance of combining vaccination with management and biosafety measures on farms to reduce *Cb* infection as well as the risks to humans, with long-term programs due to the persistence of environmental contamination by *Cb*. Finally, an improvement in standardization in studies on naturally infected Q fever in domestic ruminants is needed.

## Data availability statement

The datasets presented in this study can be found in online repositories. The names of the repository/repositories and accession number(s) can be found in the article/Supplementary material.

## Author contributions

RT-P: Conceptualization, Data curation, Formal analysis, Investigation, Methodology, Validation, Writing – original draft, Writing – review & editing, Visualization. AC: Conceptualization, Data curation, Formal analysis, Investigation, Methodology, Supervision, Validation, Writing – original draft, Writing – review & editing, Funding acquisition, Project administration, Visualization. JG: Conceptualization, Writing – review & editing, Investigation, Methodology, Visualization. JQ: Conceptualization, Writing – review & editing, Investigation, Methodology, Visualization. AG-G: Investigation, Methodology, Visualization, Writing – review & editing. AS: Conceptualization, Writing – review & editing, Investigation, Methodology, Visualization. ÁG-M: Conceptualization, Data curation, Formal analysis, Funding acquisition, Investigation, Methodology, Project administration, Resources, Supervision, Validation, Visualization, Writing – original draft, Writing – review & editing.
