# Supplementary material for: Controlling Coxiella burnetii in naturally infected sheep, goats and cows, and public health implications: a scoping review
Source: Front Vet Sci. 2024 Feb 15;11:1321553. doi: 10.3389/fvets.2024.1321553 (PMC10901991; doi:10.3389/fvets.2024.1321553)
Supplement: Supplementary file 1 [file Data_Sheet_1.docx]

**Appendix 1. Search data extraction from PubMed.**

("*Coxiella burnetii*"[All Fields] OR "Q fever"[All Fields]) AND ("ruminant*"[All Fields] OR ("goats"[MeSH Terms] OR "goats"[All Fields] OR "goat"[All Fields]) OR ("caprines"[All Fields] OR "goats"[MeSH Terms] OR "goats"[All Fields] OR "caprine"[All Fields]) OR "sheep*"[All Fields] OR ("ovines"[All Fields] OR "sheep"[MeSH Terms] OR "sheep"[All Fields] OR "ovine"[All Fields]) OR "cow"[All Fields] OR ("bovin"[All Fields] OR "cattle"[MeSH Terms] OR "cattle"[All Fields] OR "bovine"[All Fields] OR "bovines"[All Fields]) OR ("cattle"[MeSH Terms] OR "cattle"[All Fields] OR "cattle s"[All Fields] OR "cattles"[All Fields])) AND ("prevent*"[All Fields] OR "prophyla*"[All Fields] OR "vaccin*"[All Fields] OR "shedd*"[All Fields] OR "control*"[All Fields] OR "strateg*"[All Fields] OR "manage*"[All Fields] OR ("disease outbreaks"[MeSH Terms] OR ("disease"[All Fields] AND "outbreaks"[All Fields]) OR "disease outbreaks"[All Fields] OR "outbreak"[All Fields] OR "epidemiology"[MeSH Subheading] OR "epidemiology"[All Fields] OR "outbreaks"[All Fields] OR "outbreak s"[All Fields]))

Translations:

goat: "goats"[MeSH Terms] OR "goats"[All Fields] OR "goat"[All Fields]

caprine: "caprines"[All Fields] OR "goats"[MeSH Terms] OR "goats"[All Fields] OR "caprine"[All Fields]

ovine: "ovines"[All Fields] OR "sheep"[MeSH Terms] OR "sheep"[All Fields] OR "ovine"[All Fields]

bovine: "bovin"[All Fields] OR "cattle"[MeSH Terms] OR "cattle"[All Fields] OR "bovine"[All Fields] OR "bovines"[All Fields]

cattle: "cattle"[MeSH Terms] OR "cattle"[All Fields] OR "cattle's"[All Fields] OR "cattles"[All Fields]

outbreak: "disease outbreaks"[MeSH Terms] OR ("disease"[All Fields] AND "outbreaks"[All Fields]) OR "disease outbreaks"[All Fields] OR "outbreak"[All Fields] OR "epidemiology"[Subheading] OR "epidemiology"[All Fields] OR "outbreaks"[All Fields] OR "outbreak's"[All Fields]

**Appendix 2.** **Data extracted**

| **Reference** | **Language** | **Length (months)** | **Country** | **Species** | **Breed** | **Aptitude** | **Production system** | **Herds** | **Total animals in herds studied** | **Animal per herd** | **Animals in treatment group** | **Animals in control group** | **Total animals studied** |
| --- | --- | --- | --- | --- | --- | --- | --- | --- | --- | --- | --- | --- | --- |
| Álvarez-Alonso et al., (2018). | EN | 7 | ES | GO | AP | DA | IO | 1 | 77 | 77 | 35 | 0 | 35 |
| Álvarez-Alonso et al., (2020). | EN | 48 | ES | SH | LA | DA | IO | 4 | 1920 | 480 | 320 | 0 | 320 |
| Anderson et al., (2015). | EN | 12 | US | GO | BO | MT | ND | 17 | ND | ND | 674 | 0 | 674 |
| Astobiza et al., (2010). | EN | 7 | ES | SH | LA | DA | IO | 1 | 494 | 494 | 60 | 26 | 86 |
| Astobiza et al., (2011a). | EN | 10 | ES | SH | LA | DA | IO | 2 | 500 | 250 | 367 | 133 | 500 |
| Astobiza et al., (2011b). | EN | 48 | ES | SH | ND | DA | IO | 1 | 408 | 408 | 226 | 40 | 266 |
| Astobiza et al., (2012). | EN | 36 | ES | SH | LA | DA | IO | 1 | 494 | 494 | 57 | 24 | 81 |
| Avbersek et al., (2019). | EN | 18 | SI | SH | IB, JB | ND | IO | 1 | 478 | 478 | 60 | 60 | 120 |
| Bauer et al., (2021). | EN | 24 | DE | GO, SH | ML, SF, TB | ND | ND | 4 | 1705 | 426,25 | 1675 | 30 | 1705 |
| Berri et al., (2005). | EN | 35 | FR | SH | PD | ND | IO | 1 | 2001 | 2001 | 1851 | 150 | 2001 |
| Boarbi et al., (2014). | EN | 49 | BE | GO | SM | DA | ND | 124 | ND | ND | ND | ND | ND |
| de Cremoux et al., (2012). | EN | ND | FR | GO | ND | DA | ND | 3 | 905 | 301,67 | 462 | 443 | 905 |
| Eibach et al., (2013). | EN | 18 | DE | SH | ML | ND | ND | 1 | 243 | 243 | 243 | 0 | 243 |
| Garcia-Ispierto et al., (2015). | EN | 30 | ES | CA | HF | DA | IN | 1 | 750 | 750 | 212 | 208 | 420 |
| Guatteo et al., (2008). | EN | 12 | FR | CA | HF, NM | DA | ND | 6 | ND | ND | 87 | 88 | 175 |
| Hogerwerf et al., (2011). | EN | 4 | NL | GO, SH | ND | DA | ND | 13 | 957 | 73,615 | 470 | 487 | 957 |
| Luoto et al., (1951). | EN | 6 | US | CA | ND | DA | IN | 2 | ND | ND | 20 | 20 | 40 |
| Luoto et al., (1952). | EN | 5 | US | CA | HF | DA | IN | 3 | 1600 | 533,33 | 147 | 139 | 286 |
| Piñero et al., (2014). | EN | 24 | ES | CA | ND | DA | IN | 1 | 289 | 289 | 392 | ND | 417 |
| Rodolakis et al., (2009). | EN | 24 | FR | CA | ND | DA | ND | 6 | ND | ND | 98 | 44 | 142 |
| Rousset et al., (2009). | EN | ND | FR | GO | ND | DA | ND | 1 | ND | ND | 51 | 59 | 110 |
| Sádecký et al., (1975a). | EN | 4 | SK | CA | SA | DA | ND | 1 | ND | ND | 10 | 4 | 14 |
| Sádecký et al., (1975b). | EN | 36 | SK | CA | SA | DA | ND | 1 | ND | ND | 38 | 12 | 50 |
| Schmittdiel et al., (1981). | GE | 2 | SK | CA | BR, FL, HF, YL | ND | ND | 17 | ND | ND | 886 | 0 | 886 |
| Sting et al., (2013). | EN | 24 | DE | GO | ND | ND | ND | 1 | 254 | 254 | 120 | 0 | 120 |
| Taurel et al., (2012). | EN | 13 | FR | CA | ND | DA | IN | 22 | ND | ND | 1135 | 631 | 1766 |
| Taurel et al., (2014). | EN | 18 | FR | CA | ND | DA | IN | 120 | ND | ND | ND | ND | ND |
| Tutusaus et al., (2014). | EN | 12 | ES | CA | HF | DA | IN | 2 | 750 | 375 | 78 | 78 | 156 |

AP (Alpine); BE (Belgium); BR (Brown Swiss); BO (Boer); CA (Cattle); CO (Cow); DA (Dairy); EN (English); FL (Fleckvieh); FR (France); GE (German); DE (Germany); GO (Goat); HF (Holstein-Friesian); IB (Istriana breed); IN (Intensive); IO (Housed in winter/outdoor rest); JB (Jezersko-Solčava breed); LA (Latxa); MT (Meat); ML (Merino Landrace Sheep); ND (Not defined); NL (Netherlands); NM (Normande); PD (Préalpes du Sud); SM (Saanen mainly); SH (Sheep); SA (Simenthal); SK (Slovakia); SI (Slovenia); PN (Small ruminant); ES (Spain); SF (Suffolk); TB (Texel); US (United States); YL (Yellow Swiss).

| **Reference** | **Type of samples** | **Diagnostic** | **Type of Infection** | **Control/Prevention measure** | **Type of vaccine employed** |
| --- | --- | --- | --- | --- | --- |
| Álvarez-Alonso et al., (2018). | AS, BT, DS, FE, MS, VS | EL, GL, MI, PCR, VL | OU | AT, CVA, HI, HM, MM, MP, PM, VC | phase I vaccine |
| Álvarez-Alonso et al., (2020). | AS, BT, DS, FE, MS, VS | EL, GN, MI, PCR, VL | CR | HM, PM | - |
| Anderson et al., (2015). | BS, FE, MS, VS | EL, PCR | OU | BU, CL, HI | - |
| Astobiza et al., (2010). | FE, MS, VS | EL, PCR | CR | AT | - |
| Astobiza et al., (2011a). | AS, BS, VS | EL, PCR | CR | VC | Coxevac® |
| Astobiza et al., (2011b). | AS, FE, DS, MS, SS, VS | PCR | CR | VC | Coxevac® |
| Astobiza et al., (2012). | BS, BT, FE, FS, MS, PS, VS | EL, PCR | OU | AT, VC | Coxevac® |
| Avbersek et al., (2019). | BD, BS, FE, MA, MS, SS | EL, PCR | CR | VC | Coxevac® |
| Bauer et al., (2021). | BS, VS | EL, PCR | OU | VC | Coxevac® |
| Berri et al., (2005). | BS, VS | EL, PCR | OU | AT, VC | Coxevac® |
| Boarbi et al., (2014). | BT | BI, EL, GN, PCR | CR | VC | Coxevac® |
| de Cremoux et al., (2012). | BS, VS | EL, PCR | OU | VC | Coxevac® |
| Eibach et al., (2013). | BS, MA, RS, VS, WO | EL, PCR | OU | AT, VC | Coxevac® |
| Garcia-Ispierto et al., (2015). | BS | EL | CR | VC | Coxevac® |
| Guatteo et al., (2008). | FE, MS, VS | EL, PCR | CR | VC | Coxevac® |
| Hogerwerf et al., (2011). | MS, UF, VS | PCR | CR | VC | Coxevac® |
| Luoto et al., (1951). | BS, MS | CFT | CR | AT | - |
| Luoto et al., (1952). | BS, MS | CFT, GPT | CR | VC | ND |
| Piñero et al., (2014). | AS, BS, BT, DS, FE, MA, MS, UF, VS | EL, GN, PCR | CR | VC | Coxevac® |
| Rodolakis et al., (2009). | BS | EL, ST | CR | VC | Coxevac® |
| Rousset et al., (2009). | MS, VS | EL, PCR | OU | VC | Coxevac® |
| Sádecký et al., (1975a). | MS, BS | MR | CR | VC | phase I vaccine |
| Sádecký et al., (1975b). | BS, MS | MR | CR | VC | phase I vaccine |
| Schmittdiel et al., (1981). | BS, MS, PS | CFT, GPT | CR | VC | phase I vaccine |
| Sting et al., (2013). | BS, PS, VS | CFT, EL, PCR | OU | HI, HM, VC | Coxevac® |
| Taurel et al., (2012). | BS, VS | EL, PCR | CR | AT, VC | Coxevac® |
| Taurel et al., (2014). | BS, BT, MS | EL, PCR | CR | AT, VC | Coxevac® |
| Tutusaus et al., (2014). | BS, CM, FE, MS, PS, VS | EL, PCR | CR | VC | Coxevac® |

AS (Aerosol samples); AT (Antibiotherapy); BD (Bedding samples); BI (Bacterial isolation); BS (Blood samples); BT (Bulk-tank milk samples); BU (Burial); CFT (Complement fixation test); CL (Culling); CM (Colostrum); CR (Chronic); CVA (Control visitors access); DS (Dust samples); EL (ELISA); FE (Faecal Samples); FS (Foetal Samples); GN (Genotyping); GPT (Guinea-pig test); HI (Herd isolation); HM (Hygiene measures); MA (Manure samples); MI (Mouse inoculation); MM (Manure management); MP (Milk pasteurization); MR (Microagglutination reaction test); MS (Milk samples); OU (Outbreak); PCR (PCR); PM (Placenta management); PS (Placental samples); RS (Rectal swab); SS (Soil Samples); ST (Skin test); UF (Uterine fluid); VC (Vaccination); VL (Vero cell culture); VS (Vaginal swab); WO (Wool sample).

| **Reference** | **Outcome summary** |
| --- | --- |
| Álvarez-Alonso et al., (2018). | The results highlighted the lack of viable *C. burnetii* 2 months after the last parturition. Farming practices such as controlling visitor access, manure, foetuses or placentas and animal transport contributed to the reduction of the risk to the local population, as no new cases were detected in the municipality. |
| Álvarez-Alonso et al., (2020). | If *C. burnetii* infection is not controlled using a combination of vaccination and implementation of adequate biosafety and managing procedures, active infection and continuous shedding of viable bacteria can persist in sheep flocks for over 5 years. |
| Anderson et al., (2015). | A protective effect was observed for goats on farms where the primary form of goat carcass disposal was burial. Culling was not recommended to control *C. burnetii* infection in infected herds. Control measures, such as a herd management plan, should be implemented prior to lifting a temporary hold or quarantine. During an abortion storm, the sale or transport of goats, particularly pregnant does, from the affected farm should be prohibited. |
| Astobiza et al., (2010). | Control of *C. burnetii* in an infected flock based on antibiotherapy (OTC) treatment is not effective. Oxytetracycline did not prevent the shedding of bacteria or limit the duration of bacterial excretion. |
| Astobiza et al., (2011a). | Optimal results of vaccination in heavily infected sheep flocks may not be obtained in a short-term period; vaccination of heavily infected commercial flocks does not seem to have a significant effect on reducing the number of shedders and the bacterial load excreted in the first year after an outbreak of abortion. It may be an effective preventive method when administered to uninfected flocks or recently infected flocks with a high percentage of susceptible animals. |
| Astobiza et al., (2011b). | The detection of positive environmental samples after 4 years of vaccination suggests that vaccination might require a long-term commitment to reduce the potential for the re-emergence of infections in sheep herds. |
| Astobiza et al., (2012). | No statistically significant differences were found between vaccinated and control groups in sheep. With vaccination over the longer term, no differences were derived from the application of antibiotics. |
| Avbersek et al., (2019). | The combination of vaccination with extensive stable cleaning and disinfection is a short-term approach to controlling Q fever. Sheep milk used for consumption is not considered the main source of *C. burnetii* infection for humans. Faeces and manure are the most common source of environmental contamination and infection in the long term. In this sense, biosafety measures contribute to the prevention of *C. burnetii* transmission by manure. |
| Bauer et al., (2021). | Is essential that the implementation of a Q fever monitoring program be based on the persistence of antibodies due to natural infection or vaccination. The phase I vaccine did not prevent shedding entirely in the next lambing season. |
| Berri et al., (2005). | The use of antibiotherapy after a sheep Q fever outbreak showed that it did not prevent further abortions or immediately suppress the shedding of the bacteria. This control measure may have long-term effects to prevent the spread of the infection. |
| Boarbi et al., (2014). | The mandatory vaccination of animals on positive farms may have contributed to the decrease of the prevalence to 2% one year later. It led to a reduction in shedding during the first year but did not seem to be maintained until about two years later. |
| de Cremoux et al., (2012). | The use of the vaccine in exposed animals did not prevent infection under a high-infection environment but led to a decrease in vaginal shedding. Vaccination induced an overall decrease in vaginal shedding levels. It should be implemented first in renewal animals, which best respond to vaccination by significantly reducing the bacterial burden and, conversely, which excrete bacteria most massively if not vaccinated. |
| Eibach et al., (2013). | Treatment of Q fever with antibiotics (oxytetracyclines) in combination with Coxevac® was able to reduce *C. burnetii* shedding, and neither further abortions nor other clinical symptoms occurred in the sheep herd, specifically by the vaginal route, even though the vaccination was not recommended in pregnancy or in acutely infected animals. Both measures should be taken into consideration in acute Q fever outbreaks to reduce or even avoid transmission to humans. |
| Garcia-Ispierto et al., (2015). | It is possible to reduce subfertility and early foetal loss in dairy cows by maintaining them on two consecutive vaccination rounds against *C. burnetii* during advanced gestation. Vaccination did not improve the conception rate. |
| Guatteo et al., (2008). | The prevalence and bacterial load showed a reduction of that in vaccinated compared with unvaccinated cows, especially in animals during their first pregnancy. Vaccinated non-pregnant cows had a five times lower probability of becoming excretory than animals that received a placebo. The vaccination of dairy cows should be performed when there is low within-herd seroprevalence in herds where the infection has not yet spread widely. |
| Hogerwerf et al., (2011). | Q fever vaccination in small ruminants reduced the prevalence and load in uterine fluid, vaginal mucus, and milk in the Netherlands. These effects were most pronounced in young, primiparous animals. It can reasonably be assumed that vaccination under field conditions contributed to the reduction of shedding of *C. burnetii* by dairy goats and sheep, which in turn may contribute to reduction of the risk for human exposure to Q fever. |
| Luoto et al., (1951). | The treatment of cows by daily intravenous injection of a total of 15 g of aureomycin over a 5-day period was not effective at eliminating *C. burnetii* from the milk of 9 of 10 infected animals within 3 days or from 5 of 7 cows tested 6 months later in this study. Similar results were obtained for the intramammary infusion of 100 mg of aureomycin in saline solution twice a day for 5 days, which failed to overcome the infection. |
| Luoto et al., (1952). | The vaccine generated some resistance to infection and reduced the excretion of *C. burnetii* in milk. Vaccination of dairy cattle against *C. burnetii* could be a feasible control method. |
| Piñero et al., (2014). | A two-year vaccination program in dairy cattle seemed to reduce vaginal excretion in uterine fluid samples. Bulk-tank milk samples were still positive at the end of the study, and environmental samples were negative in the last six months of the study period. When uninfected animals were vaccinated, none of them shed *C. burnetii* vaginally or through milk, showing effective protection by the vaccine. The combination of vaccination and culling milk shedders reduces environmental contamination and bacterial shedding. Vaccination as a control measure for Q fever in cows must be planned as a medium-long-term strategy to suppress the risks of re-infection. |
| Rodolakis et al., (2009). | A very low proportion of cows vaccinated once in infected herds need a booster during the second year. This annual booster in uninfected heifers seems very relevant. The skin test method was evaluated at least 3 days before vaccination as a method of optimizing the number of animals needing a boost in herds, in order to detect animals with sufficient cellular immunity. |
| Rousset et al., (2009). | Young non-vaccinated goats yielded a shedding level higher than the global level. Primiparous goats responded better to the vaccine. Vaccination appeared neither able to prevent infection in exposed kids nor to clear the infection in infected goats but was effective at reducing massive bacterial shedding from a heavily infected herd and thus the risk of environmental contamination and exposure. |
| Sádecký et al., (1975a). | Vaccination with phase I *C. burnetii* protected dairy cattle against Q fever and influenced the shedding in milk of previously infected dairy cows in the long term. It may serve as an efficient preventive measure to reduce the spread of Q fever among dairy cattle. |
| Sádecký et al., (1975b). | Vaccination of dairy cattle against prevents the shedding of *C. burnetii* and its dissemination. It could be a promising method to prevent infection spread. The presence of high antibody levels does not prevent massive bacterial elimination via milk or the placenta. |
| Schmittdiel et al., (1981). | Vaccination in cattle did not prevent *C. burnetii* shedding or protect humans from Q fever infections by vaccinating infected cattle does not look very promising. |
| Sting et al., (2013). | The evaluation of different Q fever preventive measures such as indoor kidding and even vaccination of pregnant goats in combination with consistent hygiene measures showed that they were suitable actions to limit the impacts of Q fever. |
| Taurel et al., (2012). | Vaccination using a phase 1 vaccine and antibiotherapy using tetracycline is associated with a decrease in shedding in dairy cows and could contribute to reducing the bacterial load generated in the environment. |
| Taurel et al., (2014). | A significant reduction in the *C. burnetii* load was observed in herds where vaccination of ≥80% of dairy cows was implemented, and the use of antibiotics was ineffective. A vaccination strategy is an interesting measure and provides evidence for decreasing the use of antibiotics in dairy cattle herds. |
| Tutusaus et al., (2014). | The vaccination of pregnant dairy cows with an inactivated *C. burnetii* phase I vaccine at the start of the third trimester of pregnancy did not reduce bacterial shedding. There is no link between vaccination and the shedding load in pregnant cows, and it could be related to hormonal changes produced in pregnancy, which could interfere with the response to vaccination. In conclusion, vaccinating all animals in herds chronically infected with *C. burnetii* may not be an effective measure to reduce shedding. |

Appendix 3. Preferred Reporting Items for Systematic reviews and Meta-Analyses extension for Scoping Reviews (PRISMA-ScR) Checklist.

| **SECTION** | **ITEM** | **PRISMA-ScR CHECKLIST ITEM** | **REPORTED**  **ON PAGE #** |
| --- | --- | --- | --- |
| **TITLE** | | | |
| Title | 1 | Identify the report as a scoping review. | 1 |
| **ABSTRACT** | | | |
| Structured summary | 2 | Provide a structured summary that includes (as applicable): background, objectives, eligibility criteria, sources of evidence, charting methods, results, and  conclusions that relate to the review questions and objectives. | 1 |
| **INTRODUCTION** | | | |
| Rationale | 3 | Describe the rationale for the review in the context of what is already known. Explain why the review  questions/objectives lend themselves to a scoping review approach. | 1-2 |
| Objectives | 4 | Provide an explicit statement of the questions and objectives being addressed with reference to their key elements (e.g., population or participants, concepts, and context) or other relevant key elements used to  conceptualize the review questions and/or objectives. | 2 |
| **METHODS** | | | |
| Protocol and registration | 5 | Indicate whether a review protocol exists; state if and where it can be accessed (e.g., a Web address); and if available, provide registration information, including the registration number. | NA |
| Eligibility criteria | 6 | Specify characteristics of the sources of evidence used as eligibility criteria (e.g., years considered, language,  and publication status), and provide a rationale. | 2-3 |
| Information sources* | 7 | Describe all information sources in the search (e.g., databases with dates of coverage and contact with authors to identify additional sources), as well as the date the most recent search was executed. | 2-3 |
| Search | 8 | Present the full electronic search strategy for at least 1  database, including any limits used, such that it could be repeated. | Appendix 1 |
| Selection of sources of evidence† | 9 | State the process for selecting sources of evidence (i.e., screening and eligibility) included in the scoping review. | 2-3 |
| Data charting process‡ | 10 | Describe the methods of charting data from the included sources of evidence (e.g., calibrated forms or forms that have been tested by the team before their use, and whether data charting was done independently or in duplicate) and any processes for obtaining and  confirming data from investigators. | 3-4 |
| Data items | 11 | List and define all variables for which data were sought and any assumptions and simplifications made. | 3 |
| Critical appraisal of individual sources of evidence§ | 12 | If done, provide a rationale for conducting a critical appraisal of included sources of evidence; describe the  methods used and how this information was used in any data synthesis (if appropriate). | NA |
| Synthesis of results | 13 | Describe the methods of handling and summarizing the data that were charted. | 2-3 |

| **SECTION** | **ITEM** | **PRISMA-ScR CHECKLIST ITEM** | **REPORTED**  **ON PAGE #** |
| --- | --- | --- | --- |
| **RESULTS** | | | |
| Selection of sources of evidence | 14 | Give numbers of sources of evidence screened, assessed for eligibility, and included in the review, with  reasons for exclusions at each stage, ideally using a flow diagram. | Appendix 1 |
| Characteristics of sources of evidence | 15 | For each source of evidence, present characteristics for which data were charted and provide the citations. | 3-6 |
| Critical appraisal within sources of evidence | 16 | If done, present data on critical appraisal of included sources of evidence (see item 12). | NA |
| Results of  individual sources of evidence | 17 | For each included source of evidence, present the  relevant data that were charted that relate to the review questions and objectives. | Appendix 2 |
| Synthesis of results | 18 | Summarize and/or present the charting results as they relate to the review questions and objectives. | 3-7 |
| **DISCUSSION** | | | |
| Summary of evidence | 19 | Summarize the main results (including an overview of concepts, themes, and types of evidence available), link to the review questions and objectives, and consider the relevance to key groups. | 7-9 |
| Limitations | 20 | Discuss the limitations of the scoping review process. | 9 |
| Conclusions | 21 | Provide a general interpretation of the results with respect to the review questions and objectives, as well  as potential implications and/or next steps. | 9 |
| **FUNDING** | | | |
| Funding | 22 | Describe sources of funding for the included sources of evidence, as well as sources of funding for the scoping  review. Describe the role of the funders of the scoping review. | 10 |

JBI = Joanna Briggs Institute; PRISMA-ScR = Preferred Reporting Items for Systematic reviews and Meta-Analyses extension for Scoping Reviews.

* Where *sources of evidence* (see second footnote) are compiled from, such as bibliographic databases, social media platforms, and Web sites.

† A more inclusive/heterogeneous term used to account for the different types of evidence or data sources (e.g., quantitative and/or qualitative research, expert opinion, and policy documents) that may be eligible in a scoping review as opposed to only studies. This is not to be confused with *information sources* (see first footnote).

‡ The frameworks by Arksey and O’Malley (6) and Levac and colleagues (7) and the JBI guidance (4, 5) refer to the process of data extraction in a scoping review as data charting*.*

§ The process of systematically examining research evidence to assess its validity, results, and relevance before using it to inform a decision. This term is used for items 12 and 19 instead of "risk of bias" (which is more applicable to systematic reviews of interventions) to include and acknowledge the various sources of evidence that may be used in a scoping review (e.g., quantitative and/or qualitative research, expert opinion, and policy document).

*From:* Tricco AC, Lillie E, Zarin W, O'Brien KK, Colquhoun H, Levac D, et al. PRISMA Extension for Scoping Reviews (PRISMAScR): Checklist and Explanation. Ann Intern Med. 2018;169:467–473. [doi: 10.7326/M18-0850.](http://annals.org/aim/fullarticle/2700389/prisma-extension-scoping-reviews-prisma-scr-checklist-explanation)
